# Skin and hard surface disinfection against *Candida auris* – What we know today

**DOI:** 10.3389/fmed.2024.1312929

**Published:** 2024-02-07

**Authors:** Soraya Omardien, Peter Teska

**Affiliations:** ^1^Diversey Holdings Ltd., Utrecht, Netherlands; ^2^Diversey Holdings Ltd., Fort Mill, SC, United States

**Keywords:** skin disinfection, hard-surface disinfection, *Candida*, *Candida auris*, yeasticidal efficacy

## Abstract

*Candida auris* has emerged as a global healthcare threat, displaying resistance to important healthcare antifungal therapies. Infection prevention and control protocols have become paramount in reducing transmission of *C. auris* in healthcare, of which cleaning and disinfection plays an important role. *Candida albicans* is used as a surrogate yeast for yeasticidal claims of disinfection products, but reports have been made that sensitivity to disinfectants by *C. auris* differs from its surrogate. In this review, we aimed to compile the information reported for products used for skin and hard surface disinfection against *C. auris* in its planktonic or biofilm form. A comparison was made with other *Candida* species, and information were gathered from laboratory studies and observations made in healthcare settings.

## 1 Introduction

The first reported case of *Candida auris* was in 2009 when it was found in the external ear canal of an inpatient from Tokyo Metropolitan Geriatric Hospital (1). However, there is evidence that *C. auris* infections occurred as early as 1996 (2). Since then, it has been reported globally (3–7) and has become a major threat for healthcare settings according to the Centers for Disease Control and Prevention (CDC) (8), European Centre for Disease Prevention and Control (ECDC) (9) and the World Health Organization (WHO) (10). The WHO places *C. auris* together with *C. albicans* on the critical priority group for healthcare (10).

The undetected spread of *C. auris* is thought to be due to misidentification using conventional phenotypic methods, as it is fairly similar to other *Candida* species (2, 11, 12). However, major advances have been made in identifying *C. auris* using selective media and molecular diagnostic tools (11, 13–17). The greatest threat that *C. auris* poses is that it has shown to be resistant to important life-saving antifungal therapies in healthcare, such as fluconazole, amphotericin B, and echinocandins (4, 18, 19). So far, five clades of *C. auris* with a common ancestor have been identified containing antimicrobial resistance mutations and have been linked with invasive infections (12).

The genetic differences observed between the clades suggests that each clade emerged independently but simultaneously at different geographic locations (4, 20). Furthermore, each clade has been found far from its point of origin in different continents (12, 20). Clade I is generally associated with ear infections and is resistant to antifungal agents to a lesser degree (12). However, Clade II, III, IV and V have all shown resistance to fluconazole, cross resistance to amphotericin B and echinocandins, and some strains are pan-resistant (12). Pan-resistance indicates that it is not susceptible to any clinically available antimicrobials. Therefore, the greatest threat that *C. auris* pose is its resistance to antifungal therapies important in healthcare.

The best solution to control *C. auris* transmission is using infection prevention and control methods, which include cleaning and disinfection of environmental surfaces and patient care equipment. In this review, the threat that *C. auris* pose to healthcare, its mechanism of persisting on skin and surfaces, and the current knowledge of cleaning and disinfection methods to control its transmission are discussed.

## 2 The threat that *C. auris* poses in healthcare facilities

When present on environmental surfaces, *C. auris* can survive in both dry and moist areas for long durations and has shown to survive up to 7 or 14 days (21, 22). When comparing survival, *C. auris* has a greater tendency to survive on surfaces than *C. albicans* but is similar to *C. parapsilosis* or *C. glabrata* (21). It appears that *Candida* species are often present on hospital environmental surfaces and have been found on floors, sinks and high touch areas, which could all be reservoirs for transmission (23). Since *C. auris* can be present on various objects for long durations, healthcare staff and patients can become contaminated after having contact with these surfaces (5). It is, therefore, essential to reduce the presence of *C. auris* in healthcare environments as the mortality rates of *C. auris* outbreaks in areas with critically ill hospitalized patients can range from 30 to 72% (24).

## 3 Mechanisms that contribute to the persistence of *C. auris* in healthcare settings

*Candida auris* differs from other *Candida* species genetically, but also shows differences in resilience and pathogenicity (4). Even though *C. auris* is considered less virulent than *C. albicans* (25), it has shown to undergo phenotypic transitions, and to form unique multicellular aggregates (26–32). It can also tolerate high salinity and temperatures up to 42°C (22, 33, 34). The ability of *C. auris* to form biofilms appear to be the main challenge that this yeast poses to healthcare as it can persist on human skin and environmental surfaces (21, 27, 35–37). It may resist removal efforts through standard cleaning and disinfection practices where the cleaning agent cannot disrupt or reach the biofilm.

The presence of *C. auris* on skin as biofilms is not unique and has been associated with other *Candida* species (38). A study conducted by Tharp et al. (39) suggested that *C. auris* is only present or occurs on an infected person’s skin, but it was unclear whether *C. auris* was causing a dysbiosis of the skin microbiome or whether it was present as a result of the dysbiosis. The persistence of *C. auris* on the skin of patients is a great concern as living cells are continuously shed from the infected patient where it can contaminate sensitive environments in healthcare (40, 41). Horton et al. (36) compared the biofilm formation characteristics of *C. auris* with *C. albicans* in a laboratory study using skin niche conditions, including porcine skin. The observations made in this study suggests that the advantage *C. auris* has compared to *C. albicans* is its ability to survive high saline conditions and environmental desiccation that might otherwise be challenging for *C. albicans*, thereby enabling its persistence in hospital environments.

The biofilm structure formed by *C. auris* seems to be similar to what is produced by other *Candida* species, which is rich in mannan-glucan polysaccharides (42, 43). The biofilm consists of multiple layers of accumulated yeast and the formed extracellular matrix traps fluconazole thereby reducing its concentration to a level where it is not efficacious anymore (42). However, this observation is not uncommon and drug tolerance has been already associated with the extracellular matrix of biofilms (44–46). The biofilm formed by *C. albicans*, *C. parapsilosis* and *C. glabrata* has all shown to negatively impact the outcome of patients (47–49).

*Candida* species, including *C. auris*, can be controlled with the correct cleaning and disinfection practices to minimize the spread of the environmental contaminants (23). Dry surface biofilms of *Staphylococcus aureu*s have shown to be resistant to important disinfectants in healthcare, i.e., sodium hypochlorite (50), and it might be similar for *C. auris*. For instance, recovery of *C. auris* aggregative cells were observed two weeks post-treatment with sodium hypochlorite (51). Using a three-dimensional biofilm model, Kean et al. (52) observed that matured *C. auris* biofilms can tolerate chlorhexidine and hydrogen peroxide treatment. The research covering the impact of disinfectants on *C. auris* planktonic cells and biofilms is limited, but what is known will be discussed in the subsequent sections.

## 4 The efficacy of skin disinfectants against *C. auris*

Infection prevention and control (IPC) protocols and screening methods are considered the best solution to reduce transmission (6), which will include skin cleaning and disinfection. For hand hygiene, the CDC recommends, among other practices, washing with soap and water and/or the use of alcohol-based hand sanitizers prior and after the use of gloves (53, 54). A similar recommendation is made by the Public Health England (PHE) and the Centre for Opportunistic, Tropical and Hospital Infections (COTHI). Currently, there are no recommended methods for decolonization or removing *C. auris* from the skin of an infected person (53). In the following sections the findings of skin cleaning and disinfection will be summarized and discussed.

### 4.1 Chlorhexidine as skin disinfectant

Chlorhexidine is a biocide often used in products targeted for skin disinfection and is known to be effective against *Candida* species (53, 55). It’s a biocide known to target the microbial cell membrane (56). Using a suspension test (EN 13624:2013), Moore et al. (57) evaluated the efficacy of chlorhexidine against *C. auris.* The authors selected two products, one containing 2% w/v chlorhexidine with 70% (v/v) isopropyl alcohol (IPA), and the other contained 4% v/v chlorhexidine only. The products were diluted by 50% to simulate the addition of water, and the contact times ranged from 30 s to 2 min (57). The outcome of the study showed that the diluted product containing chlorhexidine with IPA had a better performance after a 2 min contact time than the product containing chlorhexidine alone. The chlorhexidine together with IPA could reduce the counts of *C. auris* and *C. albicans* by ≥ 5-logs, but chlorhexidine alone could only reduce the counts of *C. auris* by ≤ 1-log even though the concentration was higher (57). Unlike *C. auris*, *C. albicans* was more sensitive to chlorhexidine alone and the log reduction ranged from 3.57-logs in clean condition to 3.36-logs in dirty conditions. The poor performance of chlorhexidine against *C. auris*, and the improvement observed when adding IPA, has been confirmed by another study conducted on porcine skin (58).

Rutala et al. (59) also confirmed the poor performance of chlorhexidine against *C. auris* and *C. albicans* when using 2% or 4% with a disk-based quantitative method and could confirm that *C. albicans* is more sensitive to chlorhexidine than *C. auris*. A product containing 1% chlorhexidine together with 61% ethyl alcohol was also tested (59), but it had a poor performance as only a 2-log reduction was observed for *C. auris* and a 1.9-log reduction for *C. albicans*. The contact time used was 1 min (59), which might be the cause for the lower performance. Taken together, these findings show that chlorhexidine is minimally efficacious against *C. auris* when used as the sole active ingredient, but when combined with alcohol the yeasticidal performance might substantially improve.

### 4.2 Alcohol-based skin disinfectants

To disinfect hands or skin to avoid the spread of *C. auris*, alcohol-based products are recommended. Alcohol is thought to target the microbial cell membrane, and essential protein, as its mode of action (56). Rutala et al. (59) made use of a disk-based quantitative carrier test with dirty conditions and used a contact time of 1 min to understand the performance of alcohol-based products against *C. auris.* The product containing 70% IPA was efficacious against both *C. auris* and *C. albicans* as a 3.8- and 4.1-log reduction was obtained, respectively. Similarly, 70% ethanol provided a 4-log reduction for *C. auris* but only a 2.5-log reduction was obtained for *C. albicans*. It seemed that ethanol was more efficacious against *C. auris* than *C. albicans* (59). Using a pig skin model, Fu et al. (60) assessed the efficacy of various alcohol-base products against *Candida* species, including *C. auris* and *C. albicans*. The authors observed that with a contact time of 1 min, alcohol-based products are sufficient to provide a 3-log reduction. This was observed when testing ethanol (54 to 66%) combined with n-propanol (9 to 11%), and when testing ethanol (75%) alone (60). The authors interestingly observed that when combining the alcohol with an additional product containing 0.1 to 0.2% p-chloroxylenol, the log reduction was similar but that the contact time was reduced to between 15 and 30 seconds (60).

### 4.3 Other products evaluated for skin disinfection

There are other products used for skin disinfection without chlorhexidine or alcohol. Povidone-iodine was tested at a 10% concentration (1% iodine) and was found to have a limited yeasticidal performance against *C. auris* as only a 2.5-log reduction was obtained (59). It also had a poor performance against *C. albicans* as only a 2.3-log reduction was observed. The contact time used was 1 min. These findings are in contrast with the observation made by Abdolrasouli et al. (61), which reported that 10% povidone-iodine provided sufficient efficacy against *C. auris* as an 8-fold reduction in growth was obtained when performing a suspension test in a 96-well microtiter plate. Moore et al. (57) used a standardized suspension test method (EN 13624:2013) and found that 10% povidone-iodine, when used at a 2 min contact time, could reduce *C. auris* and a clinically isolated *C. albicans* strain by ≥ 4-logs in both clean and dirty conditions. *C. albicans* strain ATCC 10231 typically used in EN methods, however, had only a 2.5-log reduction in clean condition and a 3-logs reduction in dirty conditions. *C. albicans* was thus less sensitive to 10% povidone-iodine (57). Iodine interacts with essential intracellular components such as proteins and nucleic acids, which leads to cell death (56)

An antiseptic containing 3% hydrogen peroxide showed to have a poor performance against *C. auris* as only a 1.4-log reduction was obtained (59). Similarly, only a 1.8-log reduction was obtained against *C. albicans*. Hydrogen peroxide is an oxidizing agent that produce free radicals which attach essential cell components for survival (56). Chloroxylenol was tested at a 1% concentration, and it was shown to have a better performance than 3% hydrogen peroxide. However, only a 2.8-log reduction was obtained against *C. auris* and a 3.9-log reduction when tested against *C. albicans* at a 1 min contact time (59). Chloroxylenol is a halophenol and its mode of action is thought to be the cell membrane (56). The results suggest that hydrogen peroxide or chloroxylenol might have a poor performance against *C. auris* when used as a skin disinfectant, but the information about the performance of these actives against *C. auris* are limited and might differ when the contact time is extended to more than 1 min.

## 5 Hard surface disinfectants tested against *C. auris*

For hard surface disinfection, the CDC suggests the use of Environmental Protection Agency (EPA)-registered products approved for healthcare and the ECDC recommends European Standards (EN)-registered products with antifungal claims (8, 9, 53, 62). The PHI suggests the use of 1,000 ppm hypochlorite (53). The COTHI suggests 1,000 ppm chlorine-releasing agents, and optionally hydrogen peroxide vapor (53). The WHO suggests cleaning with soap and water followed by disinfection with 0.1% bleach (1,000 ppm) (53). In the subsequent sections we compiled the most recent information available about the performance of hard surface disinfectants used in healthcare tested against *C. auris*.

### 5.1 Chlorine-based disinfectants

Chlorine-based disinfectants containing sodium hypochlorite or sodium dichloroisocyanurate are commonly used in healthcare settings to disinfect against methicillin-resistant *Staphylococcus aureus* (MRSA) or carbapenemase-producing bacteria (53). Chlorine-based disinfectants typically causes oxidative damage to the cell membrane, and essential intracellular components (63). A study conducted during an outbreak at a United Kingdom hospital reported that chlorine-based disinfectants were sufficient at 1,000 ppm for daily routine disinfection of patient care areas and equipment to prevent *C. auris* (53, 64), and has been recommended by the WHO, PHI and COTHI. Moore et al. (57) utilized a standardized suspension test (EN 13624:2013) and showed that 1,000 ppm chlorine is efficacious against both *C. auris* and *C. albicans* as a ≥ 4-log reduction was obtained for both clean and dirty conditions when the contact time was 5 min. Rutala et al. (59) utilized a disk-based quantitative carrier test for two chlorine-based disinfectant products. The product contained 6,700 ppm sodium hypochlorite and was diluted to 670 ppm when tested against *C. auris*. This concentration showed to be sufficient to reduce *C. auris* by 4.1-logs and *C. albicans* by 4-logs, thereby confirming that a 1,000 ppm may be sufficient to target *C. auris*. The yeasticidal performance of chlorine-based disinfectants against *C. auris* has been confirmed in various studies, such as when using the quantitative carrier disk test method (ASTM E2197-11) (65), the EPA liquid disinfection test (EPA MLB SO MB-35-00) (66), a quantitative carrier disk test method (ASTM E-2197-02) (67) in a simulated room environment test (66) and using a microdilution method (61).

*C. auris* is expected to be present on environmental surfaces as biofilms. Therefore, it is essential to understand whether disinfectants can penetrate the protective layers of the biofilms and kill viable *C. auris* present. Limited information was available about the performance of chlorine-based products against *C. auris* biofilms, and the only study found was performed by Ledwoch et al. (68). The study combined a wipe test for bacteria (ASTM E2967-15) and a dry-biofilm method to assess the efficacy of chlorine-based disinfectants against *C. auris* dry biofilms when loaded onto wipes. Two products contained chlorine dioxide (300 ppm and 1,000 ppm), five contained sodium dichloroisocyanurate (1,000 ppm and 10,000 ppm), and two products contained sodium hypochlorite (500 and 1,000 ppm). These products had the same main active ingredient but differed in formulation composition. The authors investigated whether the wipe loaded with the product would reduce initial counts of the *C. auris* dry biofilm, whether the cells are transferable through the wipe, and whether regrowth will occur after cleaning (68).

The chlorine dioxide (1,000 ppm) product performed poorly as only a 2.5-log reduction was observed, the product transferred *C. auris* after cleaning, and regrowth post-wiping was observed after 2 days (68). Only one out of the five sodium dichloroisocyanurate products reduced the *C. auris* biofilm by ≥ 4-log, showed low transferability. Regrowth of *C. auris* was observed after 4 days. This product contained the co-formulants adipic acid, sodium toluene sulfonate, and sodium-N-lauroyl sarcosinate, in addition to the 1,000 ppm sodium dichloroisocyanurate. The product containing 1,000 ppm sodium hypochlorite reduced the *C. auris* by ≥ 7-logs and none of the *C. auris* cells picked up by the wipe was transferred to a new surface. However, regrowth was observed within 2 days. It appears that chlorine-based products containing sodium dichloroisocyanurate or sodium hypochlorite at 1,000 ppm are the best performing biocidal actives against *C. auris*, but its performance is dependent on the composition of the final formulation (68).

### 5.2 Quat and Quat-alcohol-based disinfectants

Quat is favored in many healthcare facilities due to the fungicidal, bactericidal and virucidal activity of various quat-based disinfectants (53). However, laboratory studies show that quat-based disinfectants have a poor performance against *C. auris*, and it has been implied to be the cause for an outbreak in an intensive care unit (ICU) at the Oxford University Hospital in 2018 (69). Quats are cationic surfactants that destabilizes the cell membrane, and targe essential enzymes, leading to cell lysis and eventual cell death (56, 63). The performance of quat-based products was tested against *C. auris* using a disk-based quantitative carrier test with a contact time of 1 min (59). One product contained a combination of octyl decyl dimethyl ammonium chloride (ODDMAC; 6.51%); dioctyl dimethyl ammonium chloride (DODMAC; 2.604%); didecyl dimethyl ammonium chloride (DDDMAC; 3.906%) and alkyl dimethyl benzyl ammonium chloride (ADMBAC; 8.68%). The product was diluted to have a concentration of 0.08% total quat in use. The authors observed only a 1.7-log reduction for *C. auris* and 1.5-log for *C. albicans.* A quat-based product combined with an alcohol, containing 0.1% quat with 58% ethanol in use, reduced the *C. auris* counts by 3.8-log, and the *C. albicans* counts by 4.1-log (59). These findings suggested that quat on its own has a relatively poor performance against key yeasts, and that the addition of alcohol to quat improves its performance. Other studies support the poor performance of quat against *C. auris* when used at about 0.1% quat, using a quantitative carrier test (EPA MLB SOP MB-35-00) (70, 71), and ASTM E2197-11 (65), and when used in a disinfectant wipe test (72). Even quat-based products that had supported fungicidal claims against *C. albicans* failed against *C. auris* (71), which highlights that even though a formulation has fungicidal or yeasticidal claims against *C. albicans*, it might not be similarly efficacious against *C. auris*. When the products were tested at a 10 min contact time, the log reduction was still insufficient (65, 71).

The co-active used together with quat does not necessarily need to be an alcohol. For instance, Müller et al. (73) used a suspension test (EN 13624) and a surface test with mechanical action (EN 16615) to evaluate the efficacy against *C. auris* and *C. albicans* of a quat-based product that was combine with co-actives. The product tested contained 22% ADBAC/BKC, 17% 2-phenoxyethanol, 0.9% amines N-C12-C14 (even numbered)- alkyltrimethylenedi-, reaction products with chloroacetic acid. When diluted to 0.25%, the product contained 0.06% ADBAC/BKC, 0.04% phenoxylethanol and 0.003% amine. The diluted product could reduce both yeasts by ≥ 4-logs when the suspension test was used with a 2 min contact time, and when using the surface test with a 1 min contact time (73).

When testing a quat-based product against *C. auris* dry biofilm, Ledwoch et al. (68) observed that the product was able to reduce the initial *C. auris* counts of the dry biofilm by 4-logs. The sufficient yeasticidal performance observed against *C. auris* could be due to mechanical removal when using the wipe or it could be due to the higher concentration of quat used in the product. The product contained benzalkonium chloride (BAC) and polyhexamethylene biguanide (PHMB) at a total concentration of 5,000 ppm (0.5%) quat. A low concentration of DDAC was also present. In this study, the quat-based product also prevented the transferal of about 80% of the picked-up yeast to another surface and was able to prevent regrowth for 2 days after treatment.

### 5.3 Hydrogen peroxide and peracetic acid as disinfectants

Non-quat-based disinfectants used in healthcare could contain hydrogen peroxide (HP) or peracetic acid with acetic acid (PAA). Both actives are oxidizing agents that denatures proteins and increase the permeability of the cell wall (56). Cadnum et al. (65) used the quantitative carrier disk test method (ASTM E2197-11) to evaluate the performance of peroxides against *Candida* species. A product containing 1.4% hydrogen peroxide was tested at a 1 min contact time and another containing 0.5% hydrogen peroxide was tested at a 10 min contact time. A disinfectant containing 12,000 ppm peracetic acid was also tested at a 3 min contact time. All these products reduced *C. albicans*, *C. glabrata* and *C. auris* by ≥ 5-logs. A similar observation was made by Rutala et al. (59) when using a disk-based quantitative carrier test, by Haq et al. (70) and Sexton et al. (71) using the quantitative carrier test (EPA MLB SOP MB-35-00), by Voorn et al. (72) using a wipe test, and by Cadnum et al. (67) when using a quantitative carrier test. However, yeasticidal performance is formulation dependent. For example, when testing an antiseptic that contains 3% hydrogen peroxide in use, the product had a poor performance against both *C. auris* and *C. albicans* as less than 2-log reduction was obtained (59). However, a disinfectant containing 1.4% hydrogen peroxide reduced both yeasts by 4-logs. Both products were tested with a 1 min contact time (59).

Kean et al. (74) tested the performance of 2,000 ppm peracetic acid on various carrier materials and obtained a 4-logs reduction of *C. auris, C. glabrata* and *C. albicans* after a contact time of 5 min. Notably, one out of the four *C. auris* strains tested was less sensitive to peracetic acid and was only reduced by about 3-logs. The carrier materials used were polyester polymer, stainless steel and cellulose matrix. However, even though the yeast counts could be reduced sufficiently, regrowth was observed after treating the adhered *C. auris* on stainless steel or polyester. Regrowth was not observed on the porous cellulose matrix (74).

Regrowth of *C. auris* after treatment with peracetic acid-based products was also assessed against dry-biofilms by Ledwoch et al. (68). Two products were tested containing 3,500 ppm peracetic acid, which is higher than the previously mentioned study. The authors reported a ≥ 7-logs reduction of the initial *C. auris* counts when treating a dry biofilm. Regrowth of *C. auris* after cleaning was only observed after 6 days. No yeast was transferred from the wipe after cleaning, which indicates the active could kill the yeast cells removed from the surface (68). These findings suggest that peracetic acid used at the reported concentration is efficacious against *C. auris*, it could eliminate dry biofilms and prevent regrowth shortly after cleaning.

Hydrogen peroxide was also evaluated as a vapor and was used at 8 g peroxide per cubic meter room (61). The authors tested the vapor on desiccated *C. auris* cells in a 96-well microtiter plate and obtained no viable cells after the treatment time. Hydrogen peroxide vapor was also combined with a chlorine-based cleaner (1,000 ppm) as part of a regular disinfection regime, and it was observed to aid the controlling of *C. auris* during an outbreak in a UK-based hospital (64). These findings suggest that HP or PAA-based products might be an alternative to quat-based disinfectants.

### 5.4 Other products tested as hard surface disinfectants

There are other biocidal products commercially available as hard surfaces disinfectants, but information about their performance against *C. auris* is limited. An alcohol-based product containing ethanol at 12% with 17.5% propan-1-ol was tested using a suspension test (EN 13624) and a surface test with mechanical action (EN 16615) (73). *C. auris* counts could be reduced by ≥ 4-logs using both test methods (73). Glutaraldehyde was tested at 2.4% using a quantitative carrier test method (59). A contact time of 1 min was used and the product could reduce both *C. auris* and *C. albicans* by 4.1-logs. Glutaraldehyde targets the microbial cell envelope and cross-links essential macromolecules (56). Ortho-phthalaldehyde was used at 0.55% and gave a 2.3-log reduction when tested against *C. auris* and 3.8-log reduction against *C. albicans*. A diluted product containing 0.07% *o*-phenylphenol with 0.06% *p*-tertiary amylphenol reduced *C. auris* by 4.1-logs and *C. albicans* by 3.6-logs when a 1 min contact time was used (59). The results obtained after treatment with the latter two products highlighted that there could be a difference in susceptibility between the two *Candida* species.

### 5.5 Ultraviolet light (UV-C) as a non-chemical hard surface disinfection method

No-touch disinfection methods, such as UV-C, are considered beneficial for treating hospital surfaces after manual cleaning and/or disinfection has been employed as some areas might have been missed or cleaned improperly. UV-C is known to cause damage to nucleic acids in the microbial cell (75, 76). Cadnum et al. (77) tested the impact of UV-C of healthcare relevant *Candida* species in comparison with MRSA and *Clostridium difficile.* The authors found that at 1.5 meters, MRSA was more susceptible to UV-C than *C. auris*, *C. albicans* and *C. glabrata* and *C. difficile* when exposed for 10 min. *C. auris* was the least sensitive *Candida* specie tested. *C. auris* had less than a 2-log reduction, and *C. albicans* and *C. glabrata* had less than a 3-log reduction after a 10 min exposure. However, after 20 min a more than 4-log reduction was observed for all the *Candida* species tested. When an exposure time of 30 min was used, the reduction went higher than 5-logs (77). This suggests that UV-C treatment is effective against Candida, but the exposure time is longer than what is typically used for bacteria. In contrast with the observations made by Cadnum et al. (77), Fu et al. (60) could obtain ≥ 3-logs reduction for various *Candida* species, including *C. auris* and *C. albicans*, when exposed at 1 meter for 10 min and 2.5 meter for 1 hr, which further improved when treated for a longer period (60). The *C. auris* isolates used in the study performed by Fu et al. (60) were clinical isolates from patients located in India, and Cadnum et al. (77) used clinical isolates from Germany and an isolate that showed antifungal sensitivity. The difference in observations when testing UV-C by the two separate studies could be due to the fact that different clades were being used as *C. auris* test subjects and/or the differences in UV-C doses delivered. Previous studies have reported differences in sensitivity to disinfection strategies between strains in the same clade, and between different clades (78–80), and an association between a reduced sensitivity and the aggregating phenotype has been drawn (78, 80). Overall, various studies confirmed the efficacy of UV-C against *C. auris*, but its performance depends on the dose delivered (i.e., treatment distance and exposure time) (11, 53, 81–83). The data obtained by Chatterjee et al. (78), was the only study reporting a poor performance against *C. auris* as less than 1-log reduction was obtained after a 30 min exposure time for all the clades tested.

## 6 Conclusion

Standardized biocidal efficacy methods were established to ensure that claims made by manufacturers regarding the product’s antimicrobial efficacy are reliable, reproducible and scientifically valid. These laboratory methods aim to represent “real-world” scenarios but are actually controlled conditions. “Real-world” scenarios are often complex that cannot be fully captured by standardized methods or other laboratory tests, such as whether the manufacturer’s guidelines were being followed. Therefore, these tests should be interpreted in the context of their limitations. In this review, we observed various studies showing the antimicrobial efficacy of disinfectant products against *C. auris*, amongst other *Candida* species, in laboratory settings. Limited information about the performance of these products is known in the “real-world.”

Laboratory studies suggest that quat-based products used for hard surface disinfection have limited performance against *Candida*. However, combining quat with an additional biocidal compound appears to be sufficient to improve its performance. A similar observation was made for chlorhexidine typically used for skin disinfection. Therefore, the use of quat-based products or chlorhexidine-based products should be used with the awareness that its performance depends on the formulation composition, the biocide concentration and contact time used. This point could be made of all disinfection products, irrespective of the main biocidal active present.

From studies performed on both skin and hard-surface disinfectants, it was clear that there are differences in sensitivity between *C. auris* and *C. albicans.* This difference in sensitivity was not atypical compared to other *Candida* species, such as *C. glabrata* or *C. parapsilosis.* Disinfectants with fungicidal or yeasticidal claims against *C. albicans* also showed to not be equally efficacious against *C. auris*, and differences in sensitivity between *C. auris* clades or strains were observed. These findings suggest that biocidal products should be approved for *C. auris* claims in addition to *C. albicans* when used in a healthcare setting. When testing the efficacy of a disinfectant product against *C. auris*, the clade or strain selection as a test subject should also be considered.

The unique aggregating phenotype of *C. auris* was associated with a lower sensitivity to disinfectants compared to other *Candida* species, but further research is required to understand the role that aggregation plays in the persistence or reduced sensitivity of *C. auris*. Various studies discuss the importance of biofilm formation in the persistence and transmission of *Candida*, including *C. auris*, in healthcare but only one study performed by Ledwoch et al. (68) tested various disinfectants against *C. auris* dry biofilms. Variations in efficacy between formulations with the same concentration of biocidal active ingredient was observed, but most importantly some products were unable to prevent transferring the yeast from the wipe after cleaning. Regrowth of *C. auris* after a few days of cleaning was observed and it differed between the products tested. The latter two observations are important as it might be contributing to the persistence and transmission of *C. auris* in healthcare.

In conclusion, resistance to antifungal drugs does not mean *C. auris* cannot be controlled by using cleaning and disinfection products. The findings reviewed here confirmed that efficacy still remained dependent on the composition of the formulations and not on the single biocide active present in the product. A difference in sensitivity was observed between *C. auris* and *C. albicans*, and it might benefit healthcare facilities to have a combination of both *C. auris* and *C. albicans* claims for relevant disinfection products.

## Author contributions

SO: Writing – original draft. PT: Writing – review and editing.

## References

[1] Satoh K Makimura K Hasumi Y Nishiyama Y Uchida K Yamaguchi H. Candida auris sp. nov., a novel ascomycetous yeast isolated from the external ear canal of an inpatient in a Japanese hospital. *Microbiol Immunol.* (2009) 53:41–4. 10.1111/j.1348-0421.2008.00083.x 19161556

[2] Lee WG Shin JH Uh Y Kang MG Kim SH Park KH First three reported cases of nosocomial fungemia caused by Candida auris. *J Clin Microbiol.* (2011) 49:3139–42. 10.1128/JCM.00319-11 21715586 PMC3165631

[3] Lone SA Ahmad A. Candida auris — The growing menace to global health. *Mycoses.* (2019) 62:620–37. 10.1111/myc.12904 30773703

[4] Vila T Sultan AS Montelongo-Jauregui D Jabra-Rizk MA. Candida auris: a fungus with identity crisis. *Pathog Dis.* (2020) 78:ftaa034. 10.1093/femspd/ftaa034 32643757 PMC7371155

[5] Chakrabarti A Sood P. On the emergence, spread and resistance of Candida auris: host, pathogen and environmental tipping points. *J Med Microbiol.* (2021) 70:001318. 10.1099/jmm.0.001318 33599604 PMC8346726

[6] Thatchanamoorthy N Rukumani Devi V Chandramathi S Tay ST. Candida auris: a mini review on epidemiology in healthcare facilities in Asia. *J Fungi.* (2022) 8:1126. 10.3390/jof8111126 36354893 PMC9696804

[7] Sticchi C Raso R Ferrara L Vecchi E Ferrero L Filippi D Increasing number of cases due to Candida auris in North Italy, July 2019–December 2022. *J Clin Med.* (2023) 12:1912. 10.3390/jcm12051912 36902700 PMC10003924

[8] Centers of Disease Control and Prevention. *Candida auris.* (2023). Available online at: https://www.cdc.gov/fungal/candida-auris/index.html# (accessed August 28, 2023).

[9] European Centre for Disease Prevention and Control. *Rapid Risk Assessment: Candida auris in Healthcare Settings – Europe.* (2018). Available online at: https://www.ecdc.europa.eu/en/publications-data/rapid-risk-assessment-candida-auris-healthcare-settings-europe (accessed August 28, 2023).

[10] World Health Organization. *WHO Releases First-Ever List of Health-Threatening Fungi.* (2022). Available online at: https://www.who.int/news/item/25-10-2022-who-releases-first-ever-list-of-health-threatening-fungi (accessed August 28, 2023).

[11] Chowdhary A Sharma C Meis JF. Candida auris: a rapidly emerging cause of hospital-acquired multidrug-resistant fungal infections globally. *PLoS Pathog.* (2017) 13:e1006290. 10.1371/journal.ppat.1006290 28542486 PMC5436850

[12] Sharma C Kadosh D. Perspective on the origin, resistance, and spread of the emerging human fungal pathogen Candida auris. *PLoS Pathog.* (2023) 19:e1011190. 10.1371/journal.ppat.1011190 36952448 PMC10035752

[13] Desoubeaux G Bailly É Guillaume C De Kyvon M Tellier A Morange V Candida auris in contemporary mycology labs: a few practical tricks to identify it reliably according to one recent French experience. *J Mycol Med.* (2018) 28:407–10. 10.1016/j.myc 29567284

[14] Kordalewska M Perlin DS. Identification of drug resistant Candida auris. *Front Microbiol.* (2019) 10:1918. 10.3389/fmicb.2019.01918 31481947 PMC6710336

[15] Mulet Bayona JV Salvador García C Tormo Palop N Gimeno Cardona C. Evaluation of a novel chromogenic medium for Candida spp. identification and comparison with CHROMagarTM Candida for the detection of Candida auris in surveillance samples. *Diagn Microbiol Infect Dis.* (2020) 98:115168. 10.1016/j.diagmicrobio.2020.115168 32927410

[16] Borman AM Fraser M Johnson EM. CHROMagarTM candida plus: a novel chromogenic agar that permits the rapid identification of Candida auris. *Med Mycol.* (2021) 59:253–8. 10.1093/mmy/myaa049 32525988

[17] de Jong AW Dieleman C Carbia M Mohd Tap R Hagen F. Performance of two novel chromogenic media for the identification of multidrug-resistant Candida auris compared with other commercially available formulations. *J Clin Microbiol.* (2021) 59:e03220–20. 10.1128/JCM.03220-20 33536293 PMC8092733

[18] Kilburn S Innes G Quinn M Southwick K Ostrowsky B Greenko JA Antifungal resistance trends of Candida auris clinical isolates in New York and New Jersey from 2016 to 2020. *Antimicrob Agents Chemother.* (2022) 66:e0224221. 10.1128/aac.02242-21 35007140 PMC8923207

[19] Centers for Disease Control and Prevention. *Antifungal Susceptibility Testing and Interpretation.* (2020). Available online at: https://www.cdc.gov/fungal/candida-auris/c-auris-antifungal.html (accessed August 28, 2023).

[20] Lockhart SR Etienne KA Vallabhaneni S Farooqi J Chowdhary A Govender NP Simultaneous emergence of multidrug-resistant Candida auris on 3 continents confirmed by whole-genome sequencing and epidemiological analyses. *Clin Infect Dis.* (2017) 64:134–40. 10.1093/cid/ciw691 27988485 PMC5215215

[21] Piedrahita CT Cadnum JL Jencson AL Shaikh AA Ghannoum MA Donskey CJ. Environmental surfaces in healthcare facilities are a potential source for transmission of Candida auris and other Candida species. *Infect Control Hosp Epidemiol.* (2017) 38:1107–9. 10.1017/ice.2017.127 28693657

[22] Welsh RM Bentz ML Shams A Houston H Lyons A Rose LJ Survival, persistence, and isolation of the emerging multidrug-resistant pathogenic yeast Candida auris on a plastic health care surface. *J Clin Microbiol.* (2017) 55:2996–3005. 10.1128/JCM.00921-17 28747370 PMC5625385

[23] Kumar JA Eilertson B Cadnum JL Whitlow CS Jencson AL Safdar N Environmental contamination with Candida species in multiple hospitals including a tertiary care hospital with a Candida auris outbreak. *Pathog Immun.* (2019) 4:260. 10.20411/pai.v4i2.291 31768483 PMC6827507

[24] Cortegiani A Misseri G Fasciana T Giammanco A Giarratano A Chowdhary A. Epidemiology, clinical characteristics, resistance, and treatment of infections by Candida auris. *J Intens Care.* (2018) 6:69. 10.1186/s40560-018-0342-4 30397481 PMC6206635

[25] Forgács L Borman AM Prépost E Tóth Z Kardos G Kovács R Comparison of in vivo pathogenicity of four Candida auris clades in a neutropenic bloodstream infection murine model. *Emerg Microbes Infect.* (2020) 9:1160–9. 10.1080/22221751.2020.1771218 32486923 PMC7448943

[26] Borman AM Szekely A Johnson EM. Comparative pathogenicity of United Kingdom isolates of the emerging pathogen Candida auris and other key pathogenic Candida species. *mSphere.* (2016) 1:e00189–16. 10.1128/mSphere.00189-16 27547827 PMC4990711

[27] Sherry L Ramage G Kean R Borman A Johnson EM Richardson MD Biofilm-forming capability of highly virulent, multidrug-resistant Candida auris. *Emerg Infect Dis.* (2017) 23:328–31. 10.3201/eid2302.161320 28098553 PMC5324806

[28] Yue H Bing J Zheng Q Zhang Y Hu T Du H Filamentation in Candida auris, an emerging fungal pathogen of humans: passage through the mammalian body induces a heritable phenotypic switch. *Emerg Microbes Infect.* (2018) 7:188. 10.1038/s41426-018-0187-x 30482894 PMC6258701

[29] Fan S Yue H Zheng Q Bing J Tian S Chen J Filamentous growth is a general feature of Candida auris clinical isolates. *Med Mycol.* (2021) 59:734–40. 10.1093/mmy/myaa116 33485272 PMC8257410

[30] Gao J Chow EWL Wang H Xu X Cai C Song Y LncRNA DINOR is a virulence factor and global regulator of stress responses in Candida auris. *Nat Microbiol.* (2021) 6:842–51. 10.1038/s41564-021-00915-x 34083769

[31] Santana DJ O’Meara TR. Forward and reverse genetic dissection of morphogenesis identifies filament-competent Candida auris strains. *Nat Commun.* (2021) 12:7197. 10.1038/s41467-021-27545-5 34893621 PMC8664941

[32] Bing J Guan Z Zheng T Zhang Z Fan S Ennis CL Clinical isolates of Candida auris with enhanced adherence and biofilm formation due to genomic amplification of ALS4. *PLoS Pathog.* (2023) 19:e1011239. 10.1371/journal.ppat.1011239 36913408 PMC10035925

[33] Ben-Ami R Berman J Novikov A Bash E Shachor-Meyouhas Y Zakin S Multidrug-resistant Candida haemulonii and C. auris, Tel Aviv, Israel. *Emerg Infect Dis.* (2017) 23:195–203. 10.3201/eid2302.161486 28098529 PMC5324804

[34] Rossato L Colombo AL. Candida auris: what have we learned about its mechanisms of pathogenicity? *Front Microbiol.* (2018) 9:3081. 10.3389/fmicb.2018.03081 30631313 PMC6315175

[35] Larkin E Hager C Chandra J Mukherjee PK Retuerto M Salem I The emerging pathogen Candida auris: growth phenotype, virulence factors, activity of antifungals, and effect of SCY-078, a novel glucan synthesis inhibitor, on growth morphology and biofilm formation. *Antimicrob Agents Chemother.* (2017) 61:e02396–16. 10.1128/AAC.02396-16 28223375 PMC5404565

[36] Horton MV Johnson CJ Kernien JF Patel TD Lam BC Cheong JZA Candida auris forms high-burden biofilms in skin niche conditions and on porcine skin. *mSphere.* (2020) 5:e00910–19. 10.1128/msphere.00910-19 31969479 PMC6977180

[37] Uppuluri P. Candida auris biofilm colonization on skin niche conditions. *mSphere.* (2020) 5:e00972–19. 10.1128/mSphere.00972-19 31969480 PMC6977181

[38] Kühbacher A Burger-Kentischer A Rupp S. Interaction of Candida species with the skin. *Microorganisms.* (2017) 5:32. 10.3390/microorganisms5020032 28590443 PMC5488103

[39] Tharp B Zheng R Bryak G Litvintseva AP Hayden MK Chowdhary A Role of microbiota in the skin colonization of Candida auris. *mSphere.* (2023) 8:e0062322. 10.1128/msphere.00623-22 36695588 PMC9942586

[40] Tsay S Kallen A Jackson BR Chiller TM Vallabhaneni S. Approach to the investigation and management of patients with Candida auris, an emerging multidrug-resistant yeast. *Clin Infect Dis.* (2018) 66:306–11. 10.1093/cid/cix744 29020224 PMC5798232

[41] Sexton DJ Bentz ML Welsh RM Derado G Furin W Rose LJ Positive correlation between Candida auris skin-colonization burden and environmental contamination at a ventilator-capable skilled nursing facility in Chicago. *Clin Infect Dis.* (2021) 73:1142–8. 10.1093/cid/ciab327 33978150 PMC8492228

[42] Dominguez EG Zarnowski R Choy HL Zhao M Sanchez H Nett JE Conserved role for biofilm matrix polysaccharides in Candida auris drug resistance. *mSphere.* (2019) 4:e00680–18. 10.1128/mSphereDirect.00680-18 30602527 PMC6315084

[43] Dominguez E Zarnowski R Sanchez H Covelli AS Westler WM Azadi P Conservation and divergence in the Candida species biofilm matrix mannan-glucan complex structure, function, and genetic control. *mBio.* (2018) 9:e00451–18. 10.1128/mBio.00451-18 29615504 PMC5885036

[44] Taff HT Mitchell KF Edward JA Andes DR. Mechanisms of Candida biofilm drug resistance. *Fut Microbiol.* (2013) 8:1325–37. 10.2217/fmb.13.101 24059922 PMC3859465

[45] Taff HT Nett JE Zarnowski R Ross KM Sanchez H Cain MT Biofilm-induced pathway for matrix glucan delivery: implications for drug resistance. *PLoS Pathog.* (2012) 8:e1002848. 10.1371/journal.ppat.1002848 22876186 PMC3410897

[46] Mitchell KF Zarnowski R Sanchez H Edward JA Reinicke EL Nett JE Community participation in biofilm matrix assembly and function. *Proc Natl Acad Sci USA.* (2015) 112:4092–7. 10.1073/pnas.1421437112 25770218 PMC4386410

[47] Fortún J Martín-Dávila P Gómez-García de la Pedrosa E Pintado V Cobo J Fresco G Emerging trends in candidemia: a higher incidence but a similar outcome. *J Infect.* (2012) 65:64–70. 10.1016/j.jinf.2012.02.011 22369861

[48] Rajendran R Sherry L Nile CJ Sherriff A Johnson EM Hanson MF Biofilm formation is a risk factor for mortality in patients with Candida albicans bloodstream infection—Scotland, 2012–2013. *Clin Microbiol Infect.* (2016) 22:87–93. 10.1016/j.cmi.2015.09.018 26432192 PMC4721535

[49] Soldini S Posteraro B Vella A De Carolis E Borghi E Falleni M Microbiologic and clinical characteristics of biofilm-forming Candida parapsilosis isolates associated with fungaemia and their impact on mortality. *Clin Microbiol Infect.* (2018) 24:771–7. 10.1016/j.cmi.2017.11.005 29133157

[50] Almatroudi A Gosbell IB Hu H Jensen SO Espedido BA Tahir S Staphylococcus aureus dry-surface biofilms are not killed by sodium hypochlorite: implications for infection control. *J Hosp Infect.* (2016) 93:263–70. 10.1016/j.jhin.2016.03.020 27140421

[51] Short B Brown J Delaney C Sherry L Williams C Ramage G Candida auris exhibits resilient biofilm characteristics in vitro: implications for environmental persistence. *J Hosp Infect.* (2019) 103:92–6. 10.1016/j.jhin.2019.06.006 31226270

[52] Kean R McKloud E Townsend EM Sherry L Delaney C Jones BL The comparative efficacy of antiseptics against Candida auris biofilms. *Int J Antimicrob Agents.* (2018) 52:673–7. 10.1016/j.ijantimicag.2018.05.007 29775686

[53] Ku TSN Walraven CJ Lee SA. Candida auris: disinfectants and implications for infection control. *Front Microbiol.* (2018) 9:726. 10.3389/fmicb.2018.00726 29706945 PMC5906573

[54] Caceres DH Forsberg K Welsh RM Sexton DJ Lockhart SR Jackson BR Candida auris: a review of recommendations for detection and control in healthcare settings. *J Fungi.* (2019) 5:111. 10.3390/jof5040111 31795175 PMC6958335

[55] Huang SS. Chlorhexidine-based decolonization to reduce healthcare-associated infections and multidrug-resistant organisms (MDROs): who, what, where, when, and why? *J Hosp Infect.* (2019) 103:235–43. 10.1016/j.jhin.2019.08.025 31494130

[56] McDonnell G Russell AD. Antiseptics and disinfectants: activity, action, and resistance. *Clin Microbiol Rev*. (1999) 12:147–179. 10.1128/CMR.12.1.147 9880479 PMC88911

[57] Moore G Schelenz S Borman AM Johnson EM Brown CS. Yeasticidal activity of chemical disinfectants and antiseptics against Candida auris. *J Hosp Infect.* (2017) 97:371–5. 10.1016/j.jhin.2017.08.019 28865738

[58] Johnson CJ Eix EF Lam BC Wartman KM Meudt JJ Shanmuganayagam D Augmenting the activity of chlorhexidine for decolonization of Candida auris from porcine skin. *J Fungi.* (2021) 7:804. 10.3390/jof7100804 34682225 PMC8537331

[59] Rutala WA Kanamori H Gergen MF Sickbert-Bennett EE Weber DJ. Susceptibility of Candida auris and Candida albicans to 21 germicides used in healthcare facilities. *Infect Control Hosp Epidemiol.* (2019) 40:380–2. 10.1017/ice.2019.1 30767810

[60] Fu L Le T Liu Z Wang L Guo H Yang J Different efficacies of common disinfection methods against Candida auris and other Candida species. *J Infect Public Health.* (2020) 13:730–6. 10.1016/j.jiph.2020.01.008 32005617

[61] Abdolrasouli A Armstrong-James D Ryan L Schelenz S. In vitro efficacy of disinfectants utilised for skin decolonisation and environmental decontamination during a hospital outbreak with Candida auris. *Mycoses.* (2017) 60:758–63. 10.1111/myc.12699 28872735

[62] Sears D Schwartz BS. Candida auris: an emerging multidrug-resistant pathogen. *Int J Infect Dis.* (2017) 63:95–8. 10.1016/j.ijid.2017.08.017 28888662

[63] Jones IA Joshi LT. Biocide use in the antimicrobial era: a review. *Molecules*. (2021) 26:2276. 10.3390/molecules26082276 33919993 PMC8071000

[64] Schelenz S Hagen F Rhodes JL Abdolrasouli A Chowdhary A Hall A First hospital outbreak of the globally emerging Candida auris in a European hospital. *Antimicrob Resist Infect Control.* (2016) 5:35. 10.1186/s13756-016-0132-5 27777756 PMC5069812

[65] Cadnum JL Shaikh AA Piedrahita CT Sankar T Jencson AL Larkin EL Effectiveness of disinfectants against Candida auris and other Candida species. *Infect Control Hosp Epidemiol.* (2017) 38:1240–3. 10.1017/ice.2017.162 28793937

[66] Kumar JA Cadnum JL Jencson AL Donskey CJ. Are reduced concentrations of chlorine-based disinfectants effective against Candida auris? *Am J Infect Control.* (2020) 48:448–50. 10.1016/j.ajic.2019.08.027 31604622

[67] Cadnum JL Pearlmutter BS Haq MF Jencson AL Donskey CJ. Effectiveness and real-world materials compatibility of a novel hydrogen peroxide disinfectant cleaner. *Am J Infect Control.* (2021) 49:1572–4. 10.1016/j.ajic.2021.08.008 34416312

[68] Ledwoch K Maillard J-Y. Candida auris dry surface biofilm (DSB) for disinfectant efficacy testing. *Materials.* (2018) 12:18. 10.3390/ma12010018 30577589 PMC6337396

[69] Eyre DW Sheppard AE Madder H Moir I Moroney R Quan TP A Candida auris outbreak and its control in an intensive care setting. *N Engl J Med.* (2018) 379:1322–31. 10.1056/NEJMoa1714373 30281988

[70] Haq MF Cadnum JL Pearlmutter BS Jencson AL Donskey CJ. Effectiveness of a novel 1-step cleaner and disinfectant against Candida auris. *Infect Control Hosp Epidemiol.* (2023) 44:837–9. 10.1017/ice.2022.73 35341485

[71] Sexton DJ Welsh RM Bentz ML Forsberg K Jackson B Berkow EL Evaluation of nine surface disinfectants against Candida auris using a quantitative disk carrier method: EPA SOP-MB-35. *Infect Control Hosp Epidemiol.* (2020) 41:1219–21. 10.1017/ice.2020.278 32600492

[72] Voorn MG Kelley AM Chaggar GK Li X Teska PJ Oliver HF. Contact time and disinfectant formulation significantly impact the efficacies of disinfectant towelettes against Candida auris on hard, non-porous surfaces. *Sci Rep.* (2023) 13:5849. 10.1038/s41598-023-32876-y 37037898 PMC10086017

[73] Müller P Tan CK Ißleib U Paßvogel L Eilts B Steinhauer K. Investigation of the susceptibility of Candida auris and Candida albicans to chemical disinfectants using European Standards EN 13624 and EN 16615. *J Hosp Infect.* (2020) 105:648–56. 10.1016/j.jhin.2020.05.026 32454076

[74] Kean R Sherry L Townsend E McKloud E Short B Akinbobola A Surface disinfection challenges for Candida auris: an in-vitro study. *J Hosp Infect.* (2018) 98:433–6. 10.1016/j.jhin.2017.11.015 29203448

[75] Vassal M Gomes IB Pereira AR Simões M Braga DFO Teixeira B. Combination of UVC light with antimicrobial agents for enhanced disinfection of surfaces and liquids. *J Environ Chem Eng*. (2023) 11:109639. 10.1016/j.jece.2023.109639

[76] Rastogi RP Richa Kumar A Tyagi MB Sinha RP. Molecular mechanisms of ultraviolet radiation-induced DNA damage and repair. *J Nucleic Acids*. (2010) 2010:592980. 10.4061/2010/592980 21209706 PMC3010660

[77] Cadnum JL Shaikh AA Piedrahita CT Jencson AL Larkin EL Ghannoum MA Relative resistance of the emerging fungal pathogen Candida auris and other Candida species to killing by ultraviolet light. *Infect Control Hosp Epidemiol.* (2018) 39:94–6. 10.1017/ice.2017.239 29157326

[78] Chatterjee P Choi H Ochoa B Garmon G Coppin JD Allton Y Clade-specific variation in susceptibility of Candida auris to broad-spectrum ultraviolet C light (UV-C). *Infect Control Hosp Epidemiol.* (2020) 41:1384–7. 10.1017/ice.2020.410 33046172 PMC7720409

[79] Lemons AR McClelland TL Martin SB Lindsley WG Green BJ. Inactivation of the multi-drug-resistant pathogen Candida auris using ultraviolet germicidal irradiation. *J Hosp Infect.* (2020) 105:495–501. 10.1016/j.jhin.2020.04.011 32283175 PMC7748379

[80] Pearlmutter BS Haq MF Cadnum JL Jencson AL Carlisle M Donskey CJ. Efficacy of relatively low-cost ultraviolet-C light devices against Candida auris. *Infect Control Hosp Epidemiol.* (2022) 43:747–51. 10.1017/ice.2021.206 34011417

[81] de Groot T Chowdhary A Meis JF Voss A. Killing of Candida auris by UV-C: importance of exposure time and distance. *Mycoses.* (2019) 62:408–12. 10.1111/myc.12903 30748018 PMC6850319

[82] Mariita RM Davis JH Lottridge MM Randive RV. Shining light on multi-drug resistant Candida auris: ultraviolet-C disinfection, wavelength sensitivity, and prevention of biofilm formation of an emerging yeast pathogen. *MicrobiologyOpen.* (2022) 11:e1261. 10.1002/mbo3.1261 35212481 PMC8767514

[83] Rutala WA Kanamori H Gergen MF Sickbert-Bennett EE Weber DJ. Inactivation of Candida auris and Candida albicans by ultraviolet-C. *Infect Control Hosp Epidemiol.* (2022) 43:1495–7. 10.1017/ice.2021.214 34016204

